# Oral Supplementation with Maca Improves Social Recognition Deficits in the Valproic Acid Animal Model of Autism Spectrum Disorder

**DOI:** 10.3390/brainsci13020316

**Published:** 2023-02-13

**Authors:** Pinyue Fu, Shuxin Luo, Zhongyu Liu, Kazumi Furuhara, Takahiro Tsuji, Haruhiro Higashida, Shigeru Yokoyama, Jing Zhong, Chiharu Tsuji

**Affiliations:** 1 Research Center for Child Mental Development, Kanazawa University, Kanazawa 920-8640, Japan; 2 Division of Socio-Cognitive-Neuroscience, United Graduate School of Child Development, Osaka University, Kanazawa University, Hamamatsu University School of Medicine, Chiba University and University of Fukui, Kanazawa 920-8640, Japan; 3 Physiological Department, Guangxi University of Chinese Medicine, Nanning 530011, China; 4 Department of Ophthalmology, Faculty of Medical Sciences, University of Fukui, Fukui 910-1193, Japan; 5 Life Science Innovation Center, University of Fukui, Fukui 910-1193, Japan

**Keywords:** VPA model mice, ASD model mice, maca, social recognition deficit, oxytocin

## Abstract

Autism spectrum disorder (ASD) is a congenital, lifelong neurodevelopmental disorder whose main symptom is impaired social communication and interaction. However, no drug can treat social deficits in patients with ASD, and treatments to alleviate social behavioral deficits are sorely needed. Here, we examined the effect of oral supplementation of maca (*Lepidium meyenii*) on social deficits of in utero-exposed valproic acid (VPA) mice, widely used as an ASD model. Although maca is widely consumed as a fertility enhancer and aphrodisiac, it possesses multiple beneficial activities. Additionally, it benefits learning and memory in experimental animal models. Therefore, the effect of maca supplementation on the social behavioral deficit of VPA mice was assessed using a social interaction test, a three-stage open field test, and a five-trial social memory test. The oral supplementation of maca attenuated social interaction behavior deficit and social memory impairment. The number of c-Fos-positive cells and the percentage of c-Fos-positive oxytocin neurons increased in supraoptic and paraventricular neurons of maca-treated VPA mice. These results reveal for the first time that maca is beneficial to social memory and that it restores social recognition impairments by augmenting the oxytocinergic neuronal pathways, which play an essential role in diverse social behaviors.

## 1. Introduction

Autism spectrum disorder (ASD) is a lifelong developmental disability characterized by deficits in social interaction, communication disorder, stereotype, and repetitive patterns of behavioral output [1]. According to the United States Centers for Disease Control and Prevention, approximately one in 44 children aged 8 years was estimated to have ASD in 2018 [2]. However, currently, no drug exists to treat the core symptom, deficit in social interaction and communication.

Oxytocinergic system dysfunction is implicated in the pathogenesis of neuropsychiatric disorders with social disturbances, including ASD, social anxiety disorder, and schizophrenia [3,4]. Oxytocin (OT) is produced mainly in the paraventricular and supraoptic nuclei of the hypothalamus and is released as a neurotransmitter, acting regionally as a neuromodulator in the brain [4]. The function of OT is related to social behavior, including parental behavior, bonding, sexual behavior, consolidation, social memory, stress response, anxiety, trust, and social cognition. These diverse roles are conserved in many mammalian species [4]. Such OT roles have led to the examination of the therapeutic use of OT in patients with ASD, and multiple studies reported the beneficial effects of a single dose of intranasal OT on core symptoms of ASD [5,6,7,8,9,10]. However, reports on repeated OT administration show inconsistent results [11,12,13], hindering the development of OT as an approved drug. Therefore, elucidating the mechanism of variability in the effects of repeated OT administration may lead to the development of OT-based treatments for ASD core symptoms. On the other hand, since oxytocinergic neural circuits do not act alone in regulating core symptoms-related behaviors, identifying other co-therapeutics that modulate oxytocinergic neural circuits indirectly may be as important as finding those that act directly. Using intranasal OT combined with co-therapeutics may produce synergistic and sustained effects on core symptoms of ASD. Thus, finding the co-therapeutics may be an important factor in the development of OT-based treatments.

The etiology of ASD is highly heterogeneous. Although the genetic causes are strongly correlated with ASD, there are also many cases of “idiopathic” autism with no known genetic cause, and environmental factors such as in utero exposure to poisons, pesticides, infections, and drugs such as valproic acid (VPA) can have an impact [14,15]. VPA is a widely used antiepileptic drug [15,16] and population-based studies in Denmark reported an absolute risk of 4.42% for ASD in those children exposed to VPA in utero [17]. VPA modulates neurotransmission and regulates gene expression through epigenomic chromatin reorganization by inhibiting histone deacetylase (HDAC) activity [18]. Clinical studies have shown that VPA ingestion during pregnancy is associated with an increased incidence of neural tube defects such as spina bifida and anencephaly, developmental delay, cognitive impairment, and autism [15]. Consistent with clinical reports, in utero VPA administration to rodents induces autism-like behaviors, including impaired social interaction, restrictive and repetitive behaviors, and anxiety [16]. Thus, intrauterine VPA-exposed rodents are considered to be valid animal models for drug-induced ASD. However, a limitation of the VPA rodent model is its generalizability to the ASD population [15]. Maternal administration of VPA reproduces many of the behavioral and molecular deficits of idiopathic autism, but the majority of autistic patients are not exposed to the drug prenatally. Thus, the efficacy of the VPA rodent model may be limited to autism cases exposed to drugs with HDAC inhibitory effects. Nevertheless, a potentially valuable inference can be made from the fact that different etiologies causing autism may provoke disturbances in the same molecular pathway. Additionally, the VPA rodent model has been reported to have a deficit in OT signaling, and it has been shown that intraperitoneal or intranasal administration of OT can restore behavioral deficits in VPA rodents [19,20,21,22]. Thus, VPA rodents may be a suitable model for examining the role of OT and other therapeutic agents targeting ASD.

Maca (*Lepidium meyenii*) belongs to the cruciferous family and grows at high altitudes in Peru [23]. In 2002, it was transplanted from Peru to the Yunnan Province of China [24]. It is rich in dietary fiber; has many essential amino acids and nutrients including vitamin C, copper, and iron; and its root contains bioactive compounds [23]. It is globally consumed and is popularly used as a fertility enhancer and aphrodisiac. On the other hand, with its potential to possess multi-nutritious components, it is reported to have diverse functions, including immunomodulation, antioxidant, antidepressant, antirheumatic, UV radiation protection, hepatoprotective, anti-fatigue, and neuroprotective effects [23]. Interestingly, although the mechanism of the neuronal effect of maca is unclear, the uptake of maca extract improves learning and memory in memory-impaired model mice induced by either ethanol, ovariectomy, or scopolamine [25,26,27]. However, the effects of maca on social memory impairment in neurodevelopmental disorders, including ASD, have not yet been tested.

In this study, the effects of maca on ASD animal models, in utero VPA-exposed mice, were investigated. The effect on social recognition by maca uptake with gavage was assessed using the social interaction test, a three-stage open field test, and the five-trail social recognition test. We also explored whether maca intake affects oxytocinergic signaling pathways, which play an important role in various social behaviors.

## 2. Materials and Methods

### 2.1. Animals

The pregnant ICR female mice were injected with either valproic acid (VPA, valproic acid sodium salt; Sigma, St. Louis, MO, USA, 300 mg/kg) or an equal volume of 0.9% saline at 12.5 days of gestation. Dams were kept in a single cage and allowed to raise their offspring until weaning on postnatal day 21. The animals were housed in groups of up to five per cage. The male offspring mice were used in this experiment at the age of 6–12 weeks. All animals were kept in an animal room with a 12 h:12 h light/dark (lights on at 8:00 a.m.) cycle under a temperature controlled at 23 ± 1 °C. Food and water were supplied *ad libitum*. This study was conducted following the Fundamental Guidelines for Proper Conduct of Animal Experiment and Related Activities in Academic Research Institutions under the jurisdiction of the Ministry of Education, Culture, Sports, Science and Technology of Japan. The Committee on Animal Experimentation of Kanazawa University approved the protocol (AP-173824, AP-173826).

### 2.2. Maca Preparation and Administration

Black maca was purchased from the institute of Alpine Economic Plants, Yunnan Academy of Agricultural Sciences (Lijiang, Yunnan, China). Maca was coarsely powdered, wrapped in a gauze bag, and decocted with water 10 times the weight of maca for 2.5 h three times. The extract from three decoctions was concentrated (decoction yield was 50%, i.e., 100 g of raw drug concentrated into 50 g infusion). The gavage volume was 10 mL/kg, and the maca dose was 5 g/kg (raw drug dose). Gavage was given once daily (5:00 p.m.) for 14 days in the age of 8- to 10-week-old mice housed in the group. Then, the social interaction test, 3-stage open field test, or 5-trial social memory test was conducted on the 15th day. For the social interaction and 5-trial social memory tests, grouped mice were housed separately into individual cages on the 12th day of gavage with unacquainted stranger female mice. The female mice were removed on the 14th day of the gavage.

### 2.3. Social Interaction Test

The social interaction test was performed based on the protocol of Hara et al. with some modifications [20]. For the juvenile social interaction test, 6-week-old male mice were used. The test mice were transferred to a soundproof room and maintained in their home cage for at least 90 min to habituate the environment. All tests were conducted in a clean, transparent acrylic box (60 cm × 40 cm × 25 cm) with an open top and a black bottom. The test mice were placed in an acrylic box and allowed to explore the box for 1 h. Then, a sex-, age-, and weight-matched, unfamiliar male mouse was placed in the box. The interaction test was videotaped and used for analysis. For the social interaction test in adulthood, test mice between 9 and 12 weeks old were used. The test mice raised in the group were housed separately in individual cages with unfamiliar female mice 8–10 weeks old 3 days before the behavioral test. After 2 days, the female mice were removed, and the social interaction test was conducted on a subsequent day. On the test day, test mice in their home cage were transferred to a soundproof room for habituation for at least 90 min. Then, 8–10-week-old unfamiliar female mice, never contacted before, were placed in the home cage of the test mice. The test lasted for 10 min and was videotaped for analysis. The number of mice analyzed in the interaction test of the juvenile is 18 in the control and 10 in the VPA group. The number of mice analyzed in the interaction test of adulthood is 13 in the control and 10 in the VPA group. The gavaged mice used for the analysis are 12 for the control–water and control–maca, 10 for the VPA–water, and 13 for the VPA–maca group. For the juvenile interaction test, the contact time was calculated (nose-to-nose sniffing, nose-to-anogenital sniffing, and body contact) and the following (<1 cm apart) were calculated. For the adult social interaction test, the contact time (nose-to-anogenital sniffing) and the following (<1 cm apart) time were calculated. The investigators were blinded to the treatment groups during the analysis.

### 2.4. Five-Trial Social Memory Test

The 5-trial social memory test was carried out similarly as previously reported [28]. Briefly, the test mice raised in the group were housed singly in individual cages with unfamiliar female mice, 8–10 weeks old, 3 days before the behavioral test. After 2 days, the female mice were removed, and the test was conducted on a subsequent day. On the test day, test mice in the home cage were transferred to a soundproof room for habituation for at least 90 min. Then, same, unacquainted female mice at 8–10 weeks old were placed in the test mice’s home cage for 1 min, four times. Trials were performed at 15-min intervals. In the 5th trial, another new, unacquainted female was exposed to the test mice for 1 min. The test was videotaped and later used for analysis. The number of mice analyzed in the 5-trial social memory test is 8 in the control and VPA group. The gavaged mice used for the analysis are 8 for the control–water, 10 for the control–maca, 8 for the VPA–water, and 12 for the VPA–maca group. The analysis was conducted similarly to an adult social interaction test. The investigators were blinded to the treatment groups during the analysis.

### 2.5. Three-Stage Open Field Test

The behavior test was conducted in an open field area made of wood (60 cm × 60 cm × 20 cm) as previously reported [29] and was placed in a soundproof room. The wood box space was divided into 16 squares of equal area. The four center squares were considered the center zone, and the other 12 were considered the outer zone. The center of the center zone is considered the inner zone (13.7 cm × 13.7 cm). The behavioral test had three stages: habituation, object, and social target. In the habituation stage, mice were allowed to explore freely in the box for 10 min. Then, in the object stage, a small empty cage (10 cm × 5 cm × 5 cm) with several ventilate holes was placed in the center of the center zone, and the subject mouse was allowed to explore freely for 10 min. In the social target stage, an unfamiliar male mouse of similar age was placed into a small cage (10 cm × 5 cm × 5 cm) and placed in the inner zone. The test mice were again allowed to explore freely for 10 min. The total distance and time spent in the inner and outer zone in each stage were recorded using a digital video system and analyzed automatically using ANY-maze software. The number of mice analyzed in the 3-stage open field test is 12 in the control and 11 in the VPA group. For the test using the gavaged mice, 4–8 mice were used for each group.

### 2.6. Tissue Preparation and Immunohistochemistry

Adult mice were terminally anesthetized with sodium pentobarbital (200 mg/kg body weight, i.p.) 70 min after the social interaction tests. The brain tissues were collected as described previously [30]. Briefly, mice were transcardially perfused with a heparinized (20 U/mL) 0.9% saline solution followed by 2% paraformaldehyde in 0.1 mol/L phosphate buffer (PB). The brains were collected and incubated overnight in a postfix solution (2% paraformaldehyde and 15% sucrose in 0.1 mol/L PB) at 4 °C, then transferred in a solution of 30% sucrose in 0.1 mol/L PB. The brains were immersed for at least 72 h. The brains were cut into 40 μm-thick sections using a freezing microtome.

Immunochemical staining on free-floating sections was performed as described previously [30]. Briefly, the coronal sections stored in a cryoprotectant solution (30% ethylene glycol + 20% glycerol in 0.05 mol/L sodium phosphate buffer, pH 7.3) were rinsed in PB for 5 min, three times. Sections were permeabilized in washing buffer (0.3% Triton X-100 in PB) for 20 min followed by pre-incubation in blocking solution (3% goat serum and 0.3% Triton X-100 in PB) for 1 h at room temperature (20–25 °C) Then, sections were incubated 36 h at 4 °C with a rabbit anti-c-Fos polyclonal antibody (1:1000, 226003, Synaptic Systems, Göttingen, Germany) and mouse anti-OT monoclonal antibody (1:200, PS38, CRL-1950, American Type Culture Collection, Manassas, VA, USA) diluted in the blocking solution. After washing with washing buffer for 5 min five times, the sections were incubated with goat anti-mouse IgG antibody conjugated with Alexa Fluor 488 (1:200, A-11001, Thermo Fisher Scientific, Waltham, MA, USA), goat anti-rabbit IgG antibody conjugated with Alexa Fluor 594 (1:200, A-11012, Thermo Fisher Scientific, Waltham, MA, USA), and 4′, 6-diamidino-2-phenylindole (DAPI, 1:2000, D523, Dojindo, Kumamoto, Japan) diluted in PB for 60 min at room temperature (20–25 °C). The sections were rinsed with PB for 5 min, three times, and mounted with PermaFluor Aqueous Mounting Medium (TA-030-FM, Thermo Scientific, Kalamazoo, MI, USA) on glass slides.

### 2.7. Microscope and Analysis

Acquisition and analysis of images were performed as previously described [30]. Briefly, the Olympus IX71 inverted microscope equipped with a cooled CCD camera (Cool SNAP HQ2, Roper Scientific, Tucson, AZ, USA) was used to obtain images. According to the atlas of Franklin and Paxinos (1997), bregma between −0.58 mm~−0.94 mm, anterior–posterior axis was used for quantification of the supraoptic nucleus and paraventricular nucleus of the hypothalamus. Using ImageJ (NIH, Bethesda, MD, USA), the number of c-Fos immuno-positive nuclei in each brain section was counted and normalized to the area used for quantification. The counts were averaged from three brain sections to obtain values for each mouse. The number of mice analyzed in each group was 8–12. The percentage of c-Fos-positive OT neurons was obtained by dividing the number of c-Fos-positive OT neurons by the total number of OT neurons. All quantification was performed by blinded investigators.

### 2.8. Statistics

Prism 8 software (GraphPad Software Inc., San Diego, CA, USA) was used for statistical analysis. The data are shown as mean ± SEM. Unpaired or paired Student’s *t*-tests were used to analyze between groups or in the social interaction test, respectively. To analyze the effects of groups (control or VPA) × treatment (water or maca), two-way analysis of variance (ANOVA) was used. Two-way repeated measures ANOVA was used for either the effects of groups (control or VPA) × trials or groups (control–water, control–maca, VPA–water, VPA–maca) × trials, followed by *post hoc* analysis with Tukey’s multiple comparison test.

## 3. Results

### 3.1. Social Behavior Impairment in VPA Mice

**Figure 1:**
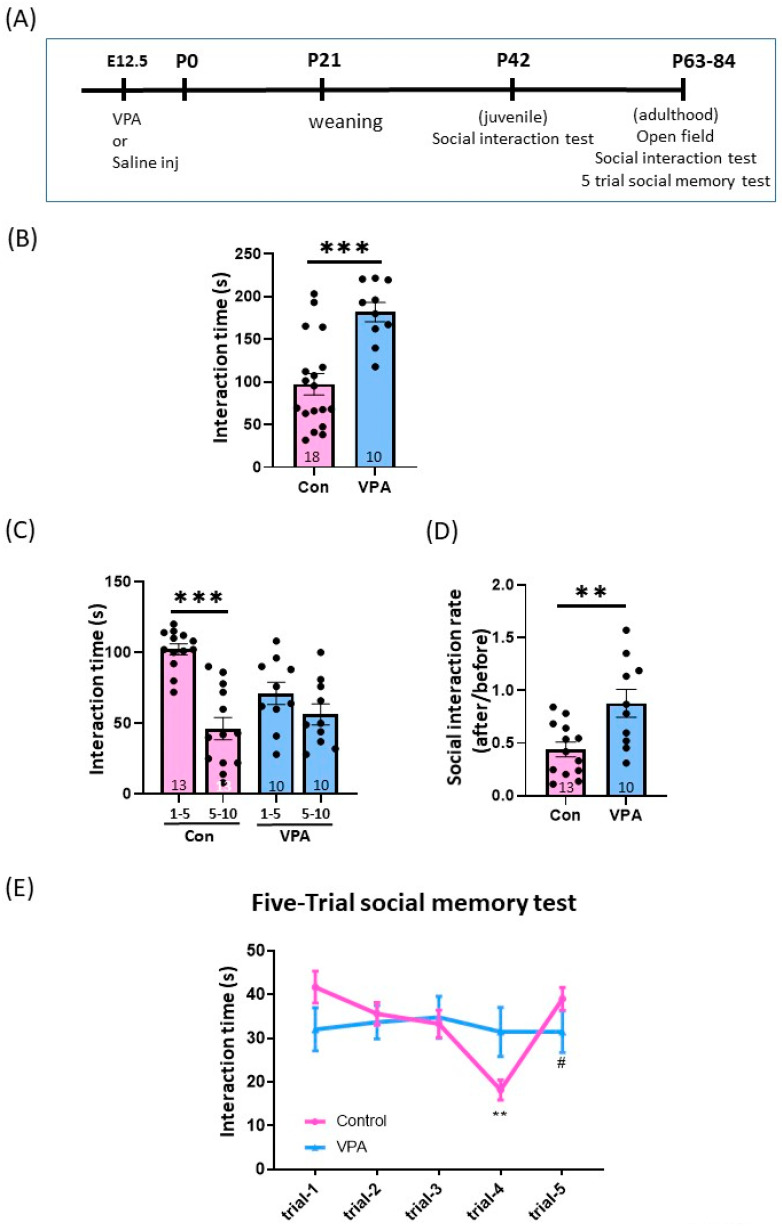
VPA-exposed animals show social interaction and social recognition memory deficit. (**A**) Experimental design. Pregnant dams were injected with either saline or valproic acid (VPA) on gestational day 12.5. The male offspring were tested in juvenile (P42) or adulthood (P63–84). (**B**) Time interacted with unfamiliar juvenile male mice during the juvenile interaction test (of in utero-exposed saline (control) or VPA mice (VPA). (**C**,**D**) Adult interaction test. (**C**) The time interacting with an unfamiliar female mouse in the first and last 5 min of the test was analyzed. (**D**) The social interaction rate. The rate was calculated as the ratio of the interaction time with unfamiliar mice in the last 5 min to the first 5 min of the test. (**E**) Interaction time in 5-trial social memory test of control and VPA mice. The same unfamiliar female mouse was used for trials 1–4, and another new female mouse was used in trial 5 (control, *n* = 8, VPA, *n* = 8). # indicates significance between trial 4 vs. trial 5, whereas * indicates significance between trials 1 and 4. The numbers of mice used in each experiment are indicated in the graphs (**B**–**E**). Data are the mean ± SEM. ** *p* < 0.01, *** *p* < 0.001, ^#^ *p* < 0.05. Con: control. VPA: valproic acid.

The in utero-exposed valproic acid (VPA) mice are a well-known animal model for ASD, showing social behavioral impairment. The social behavior of laboratory animals is assessed as the behavioral response to an unknown conspecific intruder in the home cage or a neutral test apparatus [31]. Most commonly, the shorter interaction time of the test mice with the intruder compared to the control group is considered a sign of impairment. However, impaired memory and/or increased anxiety may also be detected. Male offspring born to mothers, injected with VPA (VPA) or saline (control) on the gestational day (GD) 12.5, were assessed for their sociability in juvenile (postnatal day (PD) 42, Figure 1B) and adulthood (PD 63–84, Figure 1C,D). The juvenile male mice were first assessed in the social interaction test. VPA mice spent significantly more time contacting stranger juvenile male mice than the control mice (Figure 1B, *p* < 0.001, unpaired Student’s *t*-test). Since the total interaction time in VPA mice was higher in juveniles (Figure 1B), we suspected that VPA mice have impaired recognition memory; thus, they do not lose interest in the unfamiliar mice compared with the control mice. Acquisition of social memory has been assumed from a decline in the olfactory investigation of conspecific intruders during repeated or prolonged encounters [28,32,33,34]. Therefore, for the social interaction test using females in adulthood, we analyzed the interaction time by dividing the 10 min test window into half: the first and last parts of the test. As hypothesized, the contact time in the last 5 min was significantly lower than the first 5 min of the test in control mice (first five vs. last five, 102.2 ± 3.86 s vs. 46.31 ± 7.83 s, mean ± SEM, *p* < 0.001, paired Student’s *t*-test). Conversely, VPA mice’s contact times were similar between the two and showed no significant decrease (first 5 vs. last 5 min, 71.30 ± 7.94 vs. 56.30 ± 7.46 s, *p* = 0.168, paired student’s *t*-test, Figure 1C). The interacting ratio was significantly higher in VPA mice (Figure 1D, the last 5 min divided by the first 5 min of the test, *p* = 0.005, unpaired Student’s *t*-test). Since exposure to the unknown did not decrease the investigating time in the last part of the test in VPA mice, similar to the control mice, we further conducted five-trial social memory tests to examine whether VPA mice have impaired social acquisition and recognition (Figure 1E). The control male mice showed a characteristic decline in the time spent investigating a female during repeated pairings with the same stranger female mouse, with a full recovery following the introduction of another new stranger female in the fifth exposure. In contrast, VPA mice exhibited no decline and sustained high levels of investigation time in repeated exposure to the same female. A two-way repeated measure of ANOVA revealed a significant main effect of the group (F (4, 69) = 2.847, *p* = 0.030) and interaction (groups × trials, F (4, 69) = 2.567, *p* = 0.046). *Post hoc* Tukey’s multiple comparison tests revealed a significant difference between trial 1 and trial 4 (*p* = 0.003) as well as trial 4 and trial 5 (*p* = 0.021) in control mice, but no significance between trials was found in VPA mice. These results suggested that male VPA mice have social acquisition and recognition deficits.

### 3.2. Locomotion and Anxiety-Related Behavior in a New Environment in the Open Field Test

**Figure 2:**
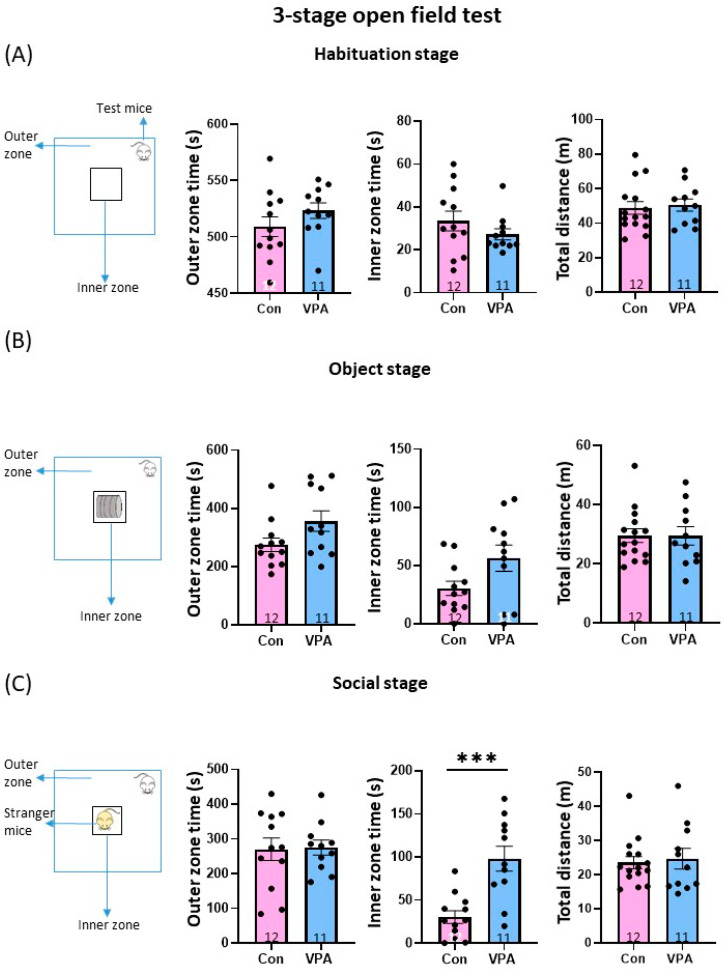
Behavior in the 3-stage open field test. The time in the outer zone, center zone, and total distance were analyzed in control and VPA mice (**A**–**C**). (**A**) Habituation stage. Behavior in the open field test with no object placed in the center. Time in the outer zone (**left**), inner zone (**middle**), and the total distance (**right**) were measured. (**B**) Object stage. Behavior in the open field with the object (food cage). (**C**) Social stage. Behavior in the open field with the male mice placed in the center. VPA mice spent more time in the inner zone than the control mice. The numbers of mice used in each experiment are indicated in the graphs. Data are the mean ± SEM. *** *p* < 0.001. Con: control. VPA: valproic acid.

The locomotor activity and social behavior was measured in a three-stage open field test as previously reported [29]. The behavioral test contained three stages: habituation, object, and social stage. Anxiety-related behavior was examined in the habituation stage of the open field test, with mice being exposed to a new environment. There was no difference between the control and VPA mice in the time spent in the inner and outer zones or traveled distance (Figure 2A). These results suggest that prenatal exposure to VPA does not affect anxiety or locomotion.

### 3.3. Anxiety-Related Behavior in the Open Field Test with a Non-Social Object

Next, we assessed the time spent in the inner and outer zones when a non-social object (empty wire cage) was placed in the center area of the open field. Similar to the habituation stage, no difference was observed between the groups in the time spent in the inner and outer zones nor traveled distance (Figure 2B). These results suggest that prenatal exposure does not affect the explorative behavior toward the object.

### 3.4. Anxiety-Related Behavior in the Open Field Test with a Social Target

Social behavior was assayed in the third stage of the open field test. The social target (an unfamiliar WT mouse of the same sex) was placed in a wire cage at the center of the arena (Figure 2C). While no significant differences were observed between the control and VPA mice in the time spent in the outer zone and the traveled distance, the time in the inner zone in the VPA mice was significantly higher than the control mice (control 30.5 ± 7.3 vs. VPA 98.2 ± 14.4 s, mean ± SEM, *p* < 0.001, unpaired Student’s *t*-tests). These results agreed with the five-trial social memory test and suggest impaired social acquisition and recognition in VPA mice.

### 3.5. Maca Attenuates the Social and Behavioral Deficit of VPA Mice

**Figure 3:**
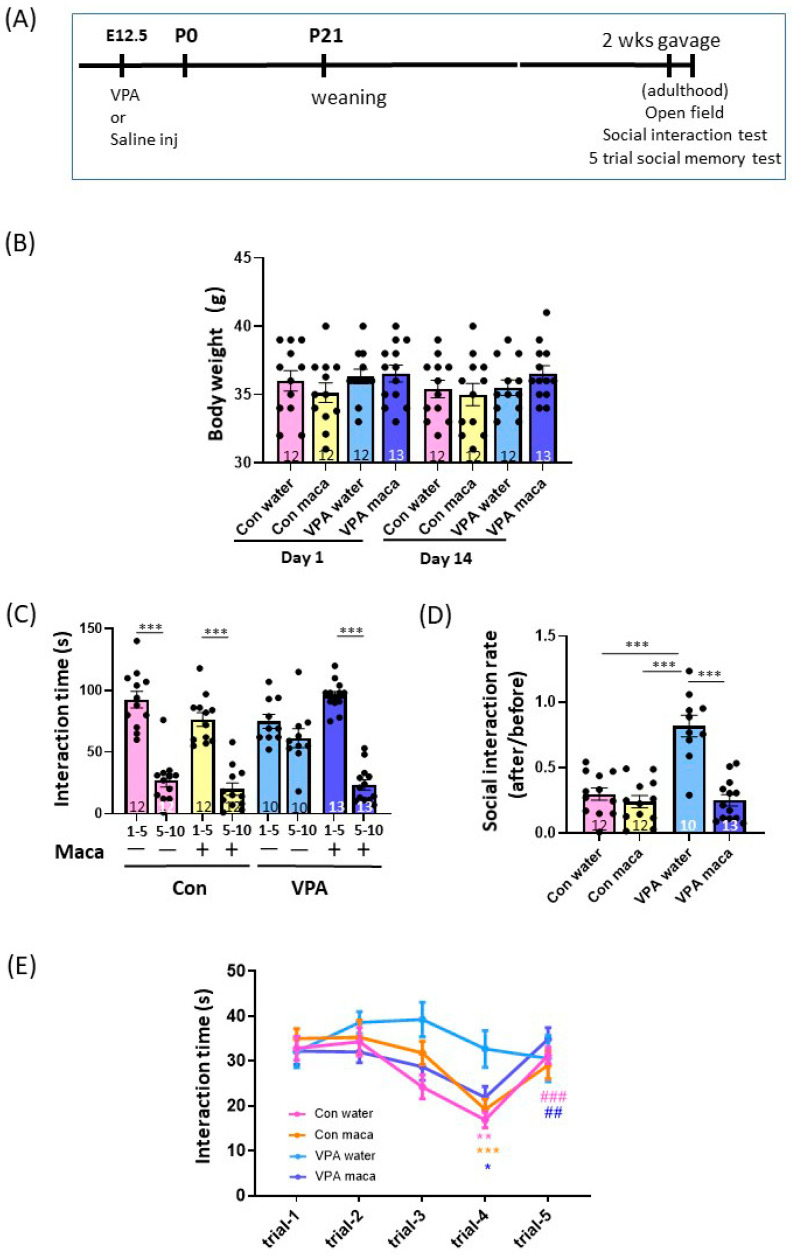
Maca improved the deficit of social behavior and social recognition memory. (**A**) Experimental design. Adult mice were gavaged with maca or water once daily for 2 weeks. Mice underwent either social interaction test, 5-trial social memory test, or an open field test after gavage. (**B**) No significant change in the weight of the mice before and after maca treatment. (**C**,**D**) The time of interaction with an unfamiliar female mouse in the first and last 5 min of the test was analyzed. (**C**) Interaction time of the first 5 and last 5 min of the test of either gavaged with water or maca. (**D**) Social interaction rate. (**E**) Interaction time in 5-trial social memory test (Con and VPA water, *n* = 8, Con maca, *n* = 10 and VPA maca, *n* = 12). # indicates significance between trial 4 vs. trial 5, whereas * indicates significance between trials 1 and 4. The number of mice used in each experiment is indicated in the graphs. Data are the mean ± SEM. * *p* < 0.05, ** *p* < 0.01, *** *p* < 0.001, ^##^ *p* < 0.01, ^###^ *p* < 0.001. Con: control. VPA: valproic acid.

The oral administration of maca improved cognitive function and spatial memory in the memory and learning deficit mice models [25,26,27,35]. We investigated whether maca uptake attenuates the social recognition memory and social behavioral deficits of VPA mice (Figure 3A). The maca or water was administered through gavage for 2 weeks in either the control (control–water, control–maca) or VPA (VPA–water, VPA–maca) group. No weight change was observed after 2 weeks of gavage in any group (Figure 3B). The maca uptake effect was first evaluated in the social interaction test. The interaction time in the control groups decreased after 5 min of the test. In contrast, while the VPA–water group interacted similarly before and after 5 min of the test, the VPA–maca group decreased significantly in the interaction after 5 min of the test (first 5 vs. last 5 min, 95.85 ± 3.31 vs. 23.38 ± 4.09 s, mean ± SEM, *p* < 0.001, paired Student’s *t*-test). The VPA–water group was similar between the two (first 5 vs. last 5 min, 74.90 ± 5.61 vs. 61.20 ± 7.7 s, mean ± SEM, *p* = 0.063, paired Student’s *t*-test, Figure 3C). The significantly high interaction rate of the VPA–water group was decreased similarly to the control level in the VPA–maca group (Figure 3D). Accordingly, a two-way ANOVA revealed a significant main effect of the group (F (1, 43) = 23.58, *p* < 0.001), the treatment (F (1, 43) = 32.44, *p* < 0.001), and the interaction between two variables (F (1, 43) = 21.69, *p* < 0.001). *Post hoc* Tukey’s multiple comparison tests revealed a significant difference between the VPA–water and other groups (Figure 3D, VPA–water vs. control–water, *p* < 0.001, vs. control–maca, *p* < 0.001, vs. VPA–maca, *p* < 0.001).

To further study the effect of maca treatment on the social acquisition and recognition, five-trial social memory tests were conducted (Figure 3E). While the VPA–water showed no decrease in the interaction time during the repeated pairing with the same females, VPA–maca showed a decline in the investigating time, with recovery following the introduction of a new female in the fifth exposure. Accordingly, two-way repeated measures of ANOVA showed a significant main effect of the interaction between the group and trial (F (12, 136) = 2.654, *p* = 0.003) and the trial (F (3.040, 103.4) = 15.80, *p* < 0.001). *Post hoc* Tukey’s multiple comparison tests revealed a significant difference between trials 1 to 4 and trials 4 to 5 in the control–water group (1 vs. 4, *p* = 0.002, 4 vs. 5, *p* < 0.001) and VPA–maca (1 vs. 4, *p* = 0.003, 4 vs. 5, *p* = 0.001). Furthermore, a significant difference was detected between trials 1 and 4 in the control–maca group (*p* < 0.001). Acquisition of social memory has been assumed from a decline in the olfactory investigation of conspecific intruders during repeated encounters with a conspecific [28,32,33,34]. Therefore, these results suggest that maca treatment improved the VPA mice’s social acquisition and recognition.

### 3.6. Maca Improved the Social Acquisition and Recognition of VPA Mice

**Figure 4:**
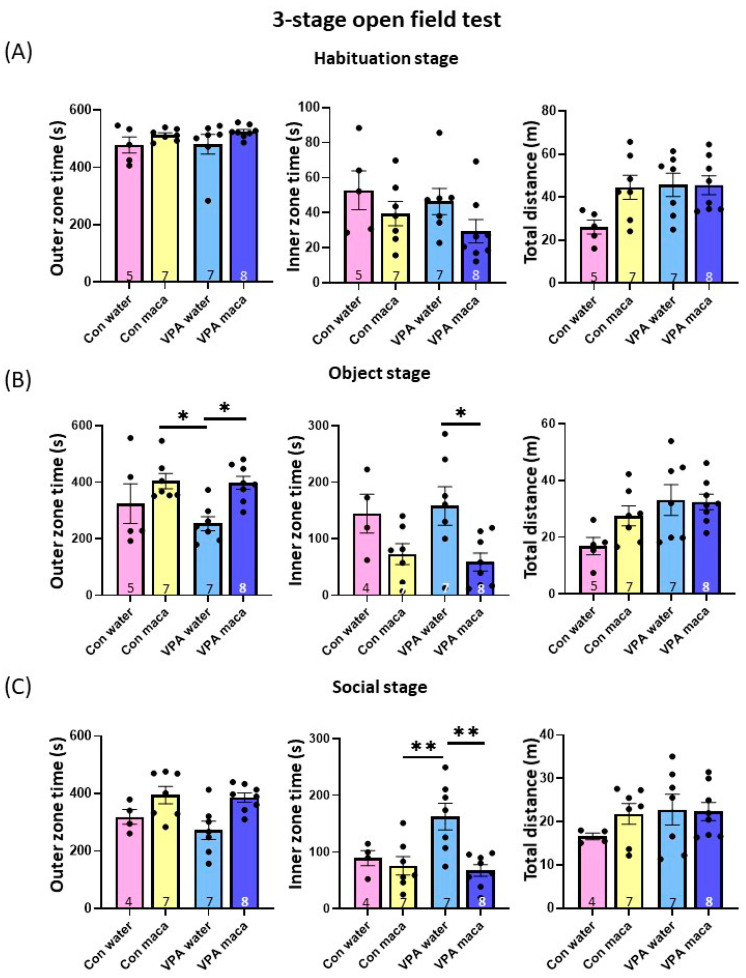
Maca recovered the social behavior deficit of VPA mice. The times in the outer zone and inner zone as well as the total distance were analyzed in the control and VPA groups gavaged with either water or maca (**A**–**C**). (**A**) Habituation stage. Behavior in the open field test with no object placed in the center. Times in the outer zone (**left**) and in center zone (**center**) as well as the total distance (**right**) were measured. (**B**) Object stage. Behavior in the open field with the object (food cage). (**C**) Social stage. Behavior in the open field with the male mice placed in the center. VPA mice spent more time in the center zone than the control mice. The number of mice used in each experiment is indicated in the graphs. Data are the mean ± SEM. * *p* < 0.05, ** *p* < 0.01. Con: control. VPA: valproic acid.

The locomotor activity and social behavior were assessed using three-stage open field tests, as in Figure 2. During the habituation stage, no significant differences existed between the groups in any index (Figure 4A). In the second stage, the times spent in the inner and outer zones were assessed when a non-social object was placed in the center area of the open field (Figure 4B). The VPA–maca group spent more time in the outer zone than the VPA–water group. Two-way ANOVA followed by *post hoc* Tukey’s multiple comparison tests showed a significant main effect of treatment (F (1, 23) = 10.16, *p* = 0.004) and revealed a significant difference between the VPA–water group and the control–maca (*p* = 0.022) and the VPA–maca group (*p* = 0.024). For the time in the inner zone, the VPA–maca spent less time there than the VPA–water group. Accordingly, two-way ANOVA followed by a *post hoc* Tukey’s multiple comparison tests exhibited a significant main effect of treatment (F (1, 22) = 10.63, *p* = 0.004) and between the VPA–water and VPA–maca groups (*p* = 0.033). No significant difference was observed between groups in the total distance. In the third stage, a social object was placed in the center area of the open field. There were no significant differences in the control and VPA groups in the time spent in the outer zone and in the total distance moved by them (Figure 4C). However, the VPA–water group spent more time in the inner zone than the other groups. Maca treatment decreased the time spent in the center for VPA mice. Accordingly, two-way ANOVA revealed a significant main effect of interaction between the group and treatment (F (1, 22) = 5.275, *p* = 0.032), and a significant main effect of treatment (F (1, 22) = 9.276, *p* = 0.006). *post hoc* Turkey’s multiple comparison tests revealed a significant difference between the VPA–water and control–maca (*p* = 0.006) and VPA–maca groups (*p* = 0.002). Furthermore, although the time in the inner zone for the VPA–water group was higher than the control–water group, it was not statistically significant (*p* = 0.061). Collectively, these data indicate that maca uptake alleviates the deficit of social acquisition and recognition in VPA mice. Additionally, maca gavage does not affect the locomotion or anxiety behavior of the control and VPA groups.

### 3.7. Maca Uptake Increased c-Fos Immunoreactivity of Oxytocinergic Cells

**Figure 5:**
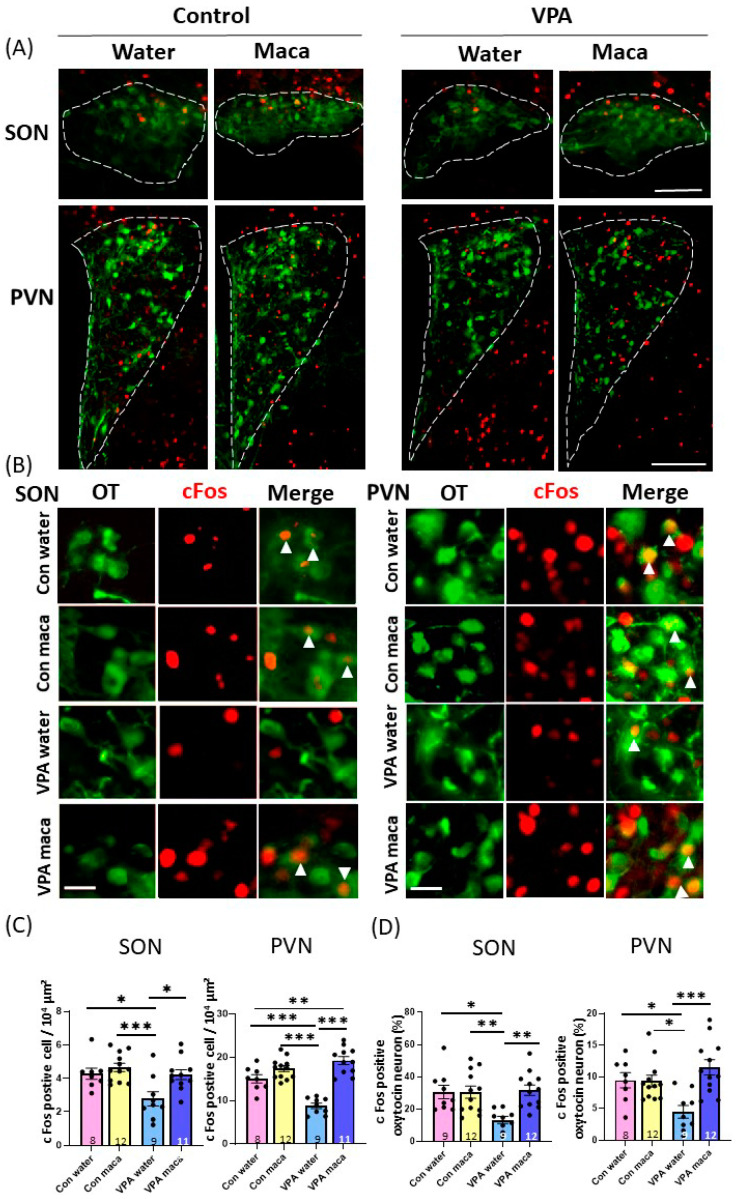
Induction of c-Fos in the supraoptic nucleus (SON) and paraventricular nucleus (PVN) of the hypothalamus after social interaction tests. (**A**) Representative merged immunostaining images in low OT magnification (green) and c-Fos (red) in the SON and PVN of control or VPA mice. The dotted areas are used for counting. Scale bars, 100 μm. (**B**) Representative immunostaining images of OT (green, (**left**)), c-Fos (red, (**middle**)), and merge (**right**) in high magnification. Scale bars, 20 μm. (**C**) The number of c-Fos-positive cells in the SON and PVN. (**D**) The percentage of c-Fos-expressing OT neurons. The percentages represent the number of c-Fos-positive OT neurons over the total number of OT neurons. The number of mice used for quantification in each group is indicated in the graph. The arrowhead shows representative c-Fos-positive OT neurons. * *p* < 0.05, ** *p* < 0.01, *** *p* < 0.001. Data are the mean ± SEM. Con: control. VPA: valproic acid.

Oxytocin (OT) is a well-known neuropeptide that regulates various social behavior [3]. It has been reported that OT administration aerolites the social and behavioral deficit of VPA mice [19,20,21,22]. We investigated the effect of maca treatment on neuronal activity of the paraventricular nucleus (PVN) and supraoptic nucleus (SON) where the OT neurons are located. The brain tissue was collected after the social interaction tests in adulthood, and immunohistochemistry was performed to detect c-Fos, a marker for neuronal activation (Figure 5A–C). The decreased number of c-Fos-positive cells in the SON of VPA mice recovered to the control group level after maca administration (Figure 5C). Two-way ANOVA showed a significant main effect of group (F (1, 36) = 8.941, *p* = 0.005), and this demonstrates a main effect of treatment (F (1, 36) = 7.849, *p* = 0.008). *Post hoc* Tukey’s multiple comparison tests revealed a significant difference in the VPA–water group and other groups (VPA–water vs. control–water, *p* = 0.02, vs. control–maca, *p* < 0.001, vs. VPA–maca, *p* = 0.015). The number of c-Fos-positive PVN neurons was also less in the VPA–water group but recovered to the control group level with maca uptake (Figure 5A–C). Accordingly, two-way ANOVA showed a significant main effect of interaction between the group and the treatment (F (1, 36) = 23.86, *p* < 0.001), a significant main effect of treatment (F (1, 36) = 7.143, *p* = 0.011), and a significant main effect of the group (F (1, 36) = 59.95, *p* < 0.001). *Post hoc* Tukey’s multiple comparison tests revealed a significant difference in the VPA–water group and other groups (VPA–water vs. control–water, *p* < 0.001, vs. control–maca, *p* < 0.001, vs. VPA–maca, *p* < 0.001). These results indicated that neuronal activation in the SON and PVN were lower in VPA mice than in the control mice. However, maca supplementation increased neuronal activation in VPA mice to levels observed in control mice.

We investigated the potential differences in the activity of OT neurons following the social interaction test by assessing the percentage of c-Fos-positive OT neurons in the SON and PVN. In both regions, the percentage of c-Fos-positive OT neurons was less in the VPA–water group, but the maca supplementation increased the percentage to the control group level (Figure 5D). Accordingly, in the SON, two-way ANOVA revealed a significant main effect of interaction between the group and treatment (F (1, 38) = 7.027, *p* = 0.012), a significant main effect of treatment (F (1, 38) = 6.748, *p* = 0.013), and a significant main effect of group (F (1, 38) = 5.223, *p* = 0.028). *Post hoc* Tukey’s multiple comparison tests revealed a significant difference between the VPA–water group and other groups (VPA–water vs. control–water, *p* = 0.012, vs. control–maca, *p* = 0.007, vs. VPA–maca, *p* = 0.004). For the PVN, two-way ANOVA revealed a significant main effect of interaction between genotype and treatment (F (1, 37) = 10.18, *p* = 0.003), a significant main effect of treatment (F (1, 37) = 9.582, *p* = 0.004). *Post hoc* Tukey’s multiple comparison tests revealed a significant difference between the VPA–water group and other groups (VPA–water vs. control–water, *p* = 0.030, vs. control–maca, *p* = 0.017, vs. VPA–maca. *p* < 0.001). According to these findings, maca treatment significantly activated OT neurons in the SON and PVN in VPA mice. Nevertheless, neuronal activation in the control mice was not affected by maca supplementation.

## 4. Discussion

In this study, we showed that maca uptake rescues the deficits of social behavior and social recognition memory in VPA mice, a mouse model of autism. The c-Fos immunoreactivity of oxytocinergic neurons in SON and PVN increased significantly after maca treatment in VPA mice. Following previous studies indicating that OT administration ameliorates the impairment of social behavior in VPA mice [19,20,21,22], maca may also have improving effects on the deficit of social behavior and social recognition memory of VPA mice, probably by activating the OT neuronal pathway. Previous studies showed that maca could improve cognitive function in the mice model of impaired cognitive memory induced by either ovariectomy, ethanol, or scopolamine [25,26,27]. Further studies are necessary to elucidate the potential link between maca and OT and to determine which components are involved in improving social recognition memory.

PVN oxytocinergic neurons are involved in diverse social behaviors including social recognition, social learning, and memory. We previously reported in other ASD model mice, CD157KO, that oral supplementation of L-carnosine, imidazole dipeptide, ameliorated deficits in social recognition via an increase in OT release [30]. We also showed that the number of c-Fos-positive cells in the PVN, SON, and basolateral amygdala (BLA) was increased after the social recognition test. OT has been reported to function on connections between the prefrontal cortex and amygdala, and this pathway is known to modulate sociality in rodents and primates, including humans [36]. In mice, the neural connection from the medial prefrontal cortex to BLA is implicated in social recognition [36], and c-Fos induction was detected in the BLA after social novelty preference experiments [37]. Furthermore, the pathway from the BLA to the infralimbic cortex or prelimbic cortex is shown to be involved in social approach-avoidance behavior using the Targeted Recombination in Active Populations method [38]. Therefore, the same neuronal pathway may be impaired in the VPA mice, and the improvement is observed in the prefrontal cortex and BLA pathway. Further study will be carried out to understand which of the neuronal circuits is recovered with maca treatment.

Here, we used black maca extract on VPA mice’s social cognitive function. Maca has about 13 varieties according to the color of the hypocotyls, ranging from white to black, and each has different biological characteristics [39]. Of these, black maca is most reported to affect cognitive function [25,26,27,35]. In the object stage of the open field test in Figure 4, VPA mice treated with black maca showed a significant decrease in the time spent in the inner zone. This may be due to memory improvement induced to lose interest in the object at earlier time points. Therefore, black maca may have improved VPA mice’s social recognition and object recognition memory. Further studies are needed to assess if maca affected the other types of memory in VPA mice.

In this study, we used an aqueous extract of maca, but the extraction method of maca differs from study to study. It is possible that the extracted components may differ depending on the extraction method. However, Rubio et al. compared the effects of hydroalcoholic and aqueous extracts and reported that both extracts improve spatial memory [25]. Therefore, at least these two extraction methods may contain the same substances that will enhance the memory deficit. A detailed analysis of the extracted components should be conducted to clarify which components have social memory-improving effects and whether or not they improve other types of memory as well.

VPA-treated rodents exhibited reduced preference or interest for social stimuli, and/or lower time spent with social stimuli than controls in many studies [16,40]. Contrarily, our VPA mice model exhibited a longer interaction time than the control mice. This behavioral deficit is confirmed with the five-trial social memory tests’ results. In this test, VPA mice did not exhibit a gradual decrease in interaction time after several trials, similar to the control mice. The acquisition of social memory was defined by a steady decrease in olfactory investigations in repeated or prolonged encounters with a conspecific [28,32,33,34]. Thus, VPA mice have a deficit in social cognitive memory, preventing them from losing interest in stranger mice, and that maca supplementation rescues this deficit.

It has been reported that the dose and timing of VPA in utero administration induce variability in ASD-like behavioral outcomes in rodents [16,40]. Previous studies have shown that doses of 300–800 mg/kg affect neurodevelopment and behavior [16,40]. Although we used a low dose (300 mg/kg), frequently used dosages are 500–800 mg/kg in rodents, and these higher dosages often give the behavioral output as a reduced preference or interest in the social stimuli [16]. On the other hand, Servadio et al. (2018) compared different VPA doses (350, 400, and 500 mg/kg) administered at gestation day 12.5 and found dose-dependent deficits in social communication and interaction in rat offspring, though no significant difference in the mean duration of social exploration in the lower dosages compared with the controls [41]. However, social behavior was not examined using several behavioral paradigms. Thus, the advantage of this study using a low VPA dose is that we established a VPA model with reduced social recognition memory, which is uncommon with other VPA models. Additionally, it is interesting to see whether other behavioral deficits that mimic the comorbid symptoms of ASD are detected in our VPA model mice and whether maca supplementation rescues these impairments.

OT plays a variety of roles in mammalian social behavior and has received interest as a potential therapy for ASD core symptoms. Clinical studies are in progress using intranasal OT administration. However, while the single dose of OT on measures of ASD core symptoms has been reported to have a beneficial effect [5,6,7,8,9,10], studies of repeated OT administration show inconsistent results [11,12,13]. Therefore, identifying the co-therapeutics or supplements that could modify the oxytocinergic neuronal pathways directly or indirectly may be important. One neuromodulatory system does not act independently of other neuromodulatory systems, and social behavior is not solely regulated by oxytocinergic neuronal systems. Identifying other co-therapeutics that indirectly modulate oxytocinergic pathways may be as important as finding the directly modifying drugs.

We have shown that maca improves the impairment of social memory and social behavioral deficits through oxytocinergic system modulation in this study. Although maca may not have an immediate effect on social behavioral deficits and takes days or weeks to demonstrate the effects, behavioral improvements, were visible regardless of the time of oral intake. The time between the very last oral intake of maca and the start of the social behavioral experiments in this study was more than 16 h. The duration of the maca’s effect on social behavioral deficits after the supplementation period is being investigated in our follow-up experiments. The possibility of the persistent effect of maca is very appealing, given that OT does not have a sustained effect due to its rapid metabolism, despite its immediate effects. Therefore, taking maca as a supplement while also receiving repeated OT treatment may have a synergistic, sustainable effect on improving social impairment in patients with ASD. Maca is already being used as a dietary supplement worldwide and has a high potential for practical applications [23].

OT increases the salience of social stimuli and modulates neural processes so that the organism can better engage and react to those stimuli [42]. The behavioral output of this increased social saliency relies highly on the context and the type of social information the organism receives. OT modulates neural circuits by making the brain more attentive to social stimuli and promoting synaptic plasticity and social learning [42]. Therefore, many researchers in OT field suggest that context-dependent OT administration is important to address the social deficit of ASD [3,43]. Young et al. note that OT administration immediately before cognitive and behavioral therapies, for example, Early Start Denver Model, might enhance the effectiveness of treatment and reduce the number of hours necessary to achieve results [43]. Socially oriented workshops and/or collaborative activities [44] may also be an efficient approach for ASD. On the other hand, taking short-acting OT in combination with a long-acting drug or supplement, e.g., maca, may enhance the effectiveness of cognitive and behavioral therapies. Stimulating the oxytocinergic pathway with OT and modulating oxytocinergic or downstream neuronal pathways related to social behaviors with maca, or a long-acting drug, may promote synaptic plasticity and social learning and strengthen the efficacy of the treatment.

The limitation of our study is that we interpreted our VPA mice as having a social memory deficit solely through the assessments of the social behavioral paradigm. However, further experiments, for example, the modified Barnes maze test and/or the novel object recognition task, are needed to determine whether our VPA mice have or do not have a general recognition dysfunction. The olfactory-guided foraging task and olfactory and acoustic habituation tasks are also necessary to clarify that the social memory deficits are not due to the olfactory and auditory impairments.

Furthermore, the dose-responsive effect on social memory impairment and the correlation between the dose and the duration necessary for the onset of behavioral improvement is not examined. The persistence of behavioral improvement after maca treatment is another interesting issue for further investigation. The evaluation of the dose, duration of administration, and the persistence of effects may provide useful information for the therapeutic use of maca.

## 5. Conclusions

This study showed for the first time that maca supplementation improves the impairment of social recognition memory in ASD model mice. We added the mechanism that social memory improvement may occur through the upregulation of oxytocinergic pathways. Maca highlights the possibility of treating social deficits sustainably in individuals with ASDs. 

## Data Availability

The data presented in this study are available on request from the corresponding author.
